# Allosteric regulation of *C. elegans* AMP-activated protein kinase

**DOI:** 10.17912/micropub.biology.000534

**Published:** 2022-03-17

**Authors:** Daniel M Scanlon, Jennifer M A Tullet

**Affiliations:** 1 1. School of Biosciences, University of Kent, Canterbury, Kent, CT2 7NZ, UK.

## Abstract

AMP-activated protein kinase (AMPK) is a key metabolic regulator which responds to changes in the AMP:ATP ratio within cells. In response to high AMP levels, AMPK promotes a metabolic shift towards increased catabolism and autophagy to restore cellular energy and maintain homeostasis. In *C. elegans,* AMPK is important for controlling a multitude of functions including metabolism, reproductive health, and lifespan. AMPK is a heterotrimeric protein consisting of α catalytic, β linker, and γ regulatory subunits. Active AMPK is characterised by phosphorylation of the α subunit. It is also regulated allosterically by the nucleotide AMP binding within the γ subunit. *C. elegans* have five different AMPKγ subunits and their primary amino acid sequence implies two different modes of AMP-binding. Modifying the ability of AMPKγ to bind adenine nucleotides could directly impact how effectively AMPK manages energy homeostasis. Despite the importance of the γ subunit, most *C. elegans* AMPK research has focused on the catalytic α subunit. Here, we genetically dissect the functional role of the different γ subunits in relation to physiology and lifespan. We show that in normal animals, three of these γ subunits ( *aakg-1, aakg-2,* and *aakg-3* ) are required for normal responses to AMP, and contribute to normal fecundity and lifespan.

**Figure 1. C. elegans AAKG isoforms contribute differentially to AMPK’s phospho-activation, and its role in lifespan and reproductive health:**
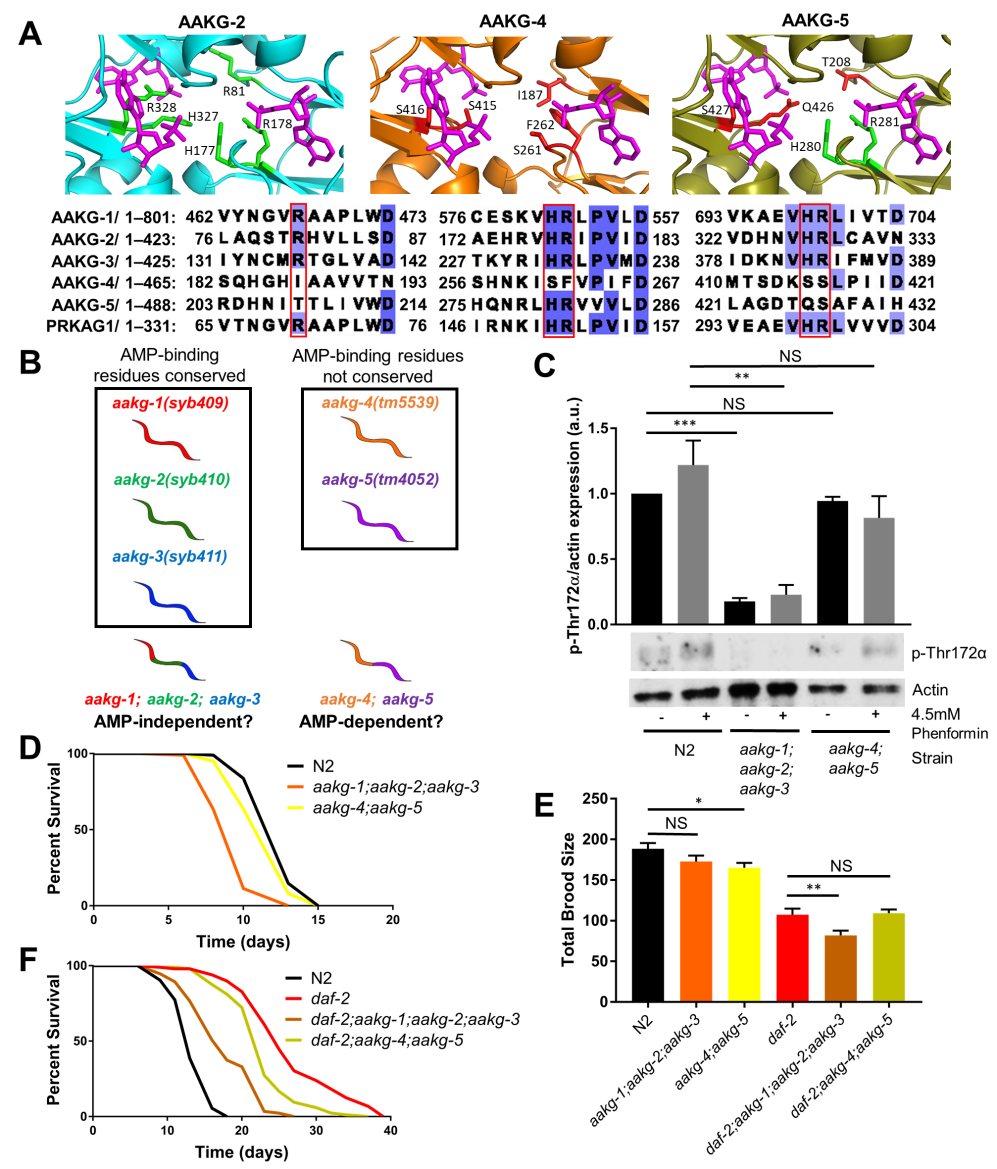
**(A)** AMP-binding sites within AAKG-2, AAKG-4, and AAKG-5 modelled using PyMOL. AAKG-1-3 show high conservation of predicted key AMP-binding residues with human PRKAG1, and AAKG-2 is shown as an example. AAKG-4 and AAKG-5 do not show conservation of AMP-binding residues with PRKAG1. For each isoform, the predicted binding residues are shown as sticks: residues in green are conserved at the functional level with PRKAG1, residues in red are not. AMP molecules are shown in magenta. A multiple sequence alignment is shown below: deeper colouring indicates higher conservation between sequences, and key predicted AMP binding residues are outlined in red. **(B)** *C. elegans* has five AMPKγ isoforms which can be grouped by the presence or absence of conserved residues predicted to influence its ability to bind AMP (as in **(A)** ). Mutant alleles of each γ isoform were combined genetically to create strains that we predict act AMP-dependently or AMP-independently. **(C)** Phosphorylation of the AAK-2 protein (α subunit) at Thr172. Western blot analysis with and without 4.5mM phenformin. Graph shows quantification from three biological replicates, representative blot shown below the graph. Data shown is normalised to untreated WT/N2 phosphorylation. **(D)** Effect of *aakg* mutation on WT lifespan. Representative plot of three biological replicates shown. *n* =>100 for each group. **(E)** Effect of *aakg* mutation on fecundity. Data pooled from three biological replicates. *n* =>27 for each group. **(F)** Effect of *aakg* mutation on the lifespan of animals with reduced insulin signalling ( *daf-2* mutants). Representative plot of four biological replicates shown. *n* =>100 for each group. In **C** and **E** , *= *p* <0.05, **= *p* <0.01, ***= *p* <0.001, using Student’s two-tailed *t* -test. Error bars indicate SEM. For **D** and **F,** log-rank test was used – see text for *p* values.

## Description

AMP-activated protein kinase (AMPK) is well-conserved throughout eukaryotes, and critically important for controlling energy balance in a wide variety of organisms (Hardie 2015; Carling 2017; Viollet and Foretz 2016). *C. elegans* has been especially useful for understanding the role of this kinase in a whole organism context, including its importance for lifespan and metabolism (Zhang et al. 2020; Viollet and Foretz 2016). AMPK is a heterotrimeric protein consisting of an α catalytic, β linker, and γ regulatory subunit. Active AMPK is characterised by phosphorylation of Thr172 on the α subunit, and in *C. elegans* research has focused on this subunit, using a mutant allele of the gene ( *aak-2* ) that removes it entirely. AMPK is regulated allosterically by the nucleotide AMP binding within the γ subunit, initiating conformational change across the kinase which both promotes phosphorylation and inhibits dephosphorylation of the α catalytic subunit (Lee et al. 2008; Hardie 2015; Carling 2017). *C. elegans* encodes five γ subunits (denoted *aakg-1-5)* which are 30-47% similar to mammalian PRKAG1 at the DNA sequence level.

The primary amino acid sequence of *C. elegans* AAKG revealed isoform-specific differences at residues required for nucleotide binding (Tullet et al. 2014). AMP-sensing relies on interaction between positively-charged amine groups and negatively-charged phosphate groups on AMP molecules (Figure 1A). Histidine and arginine residues involved in AMP binding in AAKG-1-3 are highly conserved in human PRKAG1 whereas in AAKG-4 and AAKG-5 these residues are not conserved (Figure 1A). This suggested to us that *C. elegans* encoded two isoform families of AAKG, with different sensitivities to AMP (Figure 1B).

To determine the role of these two putative AAKG isoform families in the regulation and function of AMPK, we obtained and generated a library of individual *aakg* mutants, using classical genetics to create *aakg-1; aakg-2; aakg-3* triple mutants and *aakg-4; aakg-5* double mutants. All strains were homozygous viable and appeared healthy. To investigate the impact of combining these *aakg* mutations on AMPK activation, we compared phospho-activation of the α subunit in WT *C. elegans vs* . *aakg-1; aakg-2; aakg-3* and *aakg-4; aakg-5* mutants *.* As expected, WT animals showed moderate levels of AAK-2 Thr172 phosphorylation which was moderately increased in response to the known AMPK activator phenformin (Figure 1C) (Cabreiro et al. 2013; Weir et al. 2017). However, Thr172 phosphorylation was almost undetectable in *aakg-1; aakg-2; aakg-3* triple mutants, indicating that this family of *aakg* isoforms is required to induce phosphorylation of the α subunit. In contrast, the α subunit can be phosphorylated in *aakg-4; aakg-5* double mutants. In summary, *aakg-1, aakg-2,* and *aakg-3* are required for *C. elegans* AMPK to respond effectively to AMP.

*aak-2* is required for normal lifespan (Miller et al. 2020). Individual *aakg* isoforms have also been implicated in lifespan, indicating that both catalytic and allosteric regulation of AMPK are important for longevity (Tullet et al. 2014). To test the role of AMP-binding in longevity we measured the lifespan of our *aakg-1; aakg-2; aakg-3* and *aakg-4; aakg-5* mutants compared to WT. We found that both *aakg-1; aakg-2; aakg-3 and aakg-4; aakg-5* mutants had shorter lifespans than WT (Figure 1D, *p* <0.001 and *p* <0.01, respectively), but that the effect was most pronounced in the *aakg-1; aakg-2; aakg-3* mutant. Notably, the lifespan of *aakg-1; aakg-2; aakg-3* triple mutants is similar to that reported for *C. elegans* lacking *aak-2* (Figure 1D) *,* implying that disruption of *aakg-1, -2,* and- *3* (and thereby AMP-sensitive regulation) effectively mimics the removal of the α subunit, at least in terms of WT lifespan (Apfeld et al. 2004; McQuary et al. 2016). As our *aakg* mutants were shortening lifespan, we were concerned that these mutations were making the animals sick. To assess their general health, we measured their progeny production compared to WT. However, despite its dramatic impact on lifespan, combined mutation of *aakg-1, aakg-2,* and *aakg-3* did not alter *C. elegans* brood size. In contrast, *aakg-4; aakg-5* double mutants displayed slightly reduced WT fecundity (Figure 1E, *p* <0.05). This suggests that the longevity effects are not correlated to overall health.

*aak-2* is also required for the long life incurred by genetic downregulation of the insulin signalling pathway, which is achieved in *C. elegans* through mutation of the insulin receptor *daf-2* ; and the *aak-* 2 mutation completely supresses *daf-2* longevity (Apfeld et al. 2004; Kenyon 2010; Viollet and Foretz 2016). To test the impact of AMP-binding on *daf-2* longevity we generated *daf-2; aakg-1; aakg-2; aakg-3* and *daf-2; aakg-4; aakg-5* mutants and measured their lifespan compared to *daf-2* mutation alone. Removing either family of *aakg* isoforms significantly suppressed *daf-2* longevity (Figure 1F, *p* <0.001 for both comparisons), but similarly to the situation in WT animals, the effect was most pronounced when *aakg-1, aakg-2,* and *aakg-3* were mutated. This suggests that the conserved AMP-binding region of these isoforms is important for AMPK’s contribution to *daf-2* longevity as well as that of WT. However, in this case, the suppression of *daf-2* longevity is not as complete as it is with *aak-2* mutation (Apfeld et al. 2004), indicating that both families of *aakg* isoforms have a role to play in this context. Again, we examined the health of these strains by quantifying progeny production. In contrast to the situation in WT animals, *daf-2; aakg-1; aakg-2; aakg-3* mutants exhibited slightly lower fecundity compared to *daf-2* controls (Figure 1E, *p* <0.01), whereas brood size was not altered in *daf-2; aakg-4; aakg-5* mutants (Figure 1E). This suggests that longevity and reproductive capability are not correlated in this context.

AMPK is a highly-sensitive kinase which exhibits multiple levels of regulation (Hawley et al. 1996; Oakhill et al. 2010; Xiao et al. 2013). *C. elegans* differs from mammals in that it appears to encode forms of the kinase with potential for nucleotide-independent regulation. Our data shows that deletion of three AMPKγ subunits predicted to bind AMP ( *aakg-1, aakg-2,* and *aakg-3* ) prevents phosphorylation of Thr172, an important event for AMPK activation in *C. elegans* , and this reduces WT lifespan similarly to deletion of the α subunit. We also show that removal of *aakg-1-3* does not completely suppress *daf-2* longevity; mutation of *aakg-4* and *aakg-5* also contributes here and is sufficient to alter lifespan and WT progeny production, indicating that *aakg-4* and *aakg-5* are not simply gene duplications/redundant. Mammalian AMPKγ isoforms exhibit tissue-specific expression and function (Salt et al. 1998; Thornton, Snowden, and Carling 1998; Cheung et al. 2000). *C. elegans* AMPK isoforms also exhibit tissue-specific expression ((Tullet et al. 2014), Wormbase, Version WS282). We do not know the combinations of the subunits that come together in different tissues to determine *C. elegans* lifespan and physiology, but it is conceivable that the five *aakg* isoforms could mediate AMP-independent function in specific tissues. Identifying the AMPK trimers that exist in different tissues would be informative, and provides a lucrative avenue for further study.

## Methods

***C. elegans* husbandry**

Animals grown on standard Nematode Growth Media (NGM). *E. coli* strains OP50 or OP50-1 were used as a food source (see individual protocols below). Strains were maintained at 20°C, apart from the temperature-sensitive *daf-2* ( *m577* ) strains, maintained at 15°C. All *aakg* mutations were confirmed using multiple worm PCR.

**PyMOL modelling**

Protein sequence files for each AAKG isoform were obtained from Wormbase (Version WS282). FASTA sequences were run through the Phyre2 modelling engine, and PDB files viewed in PyMOL (Version 1.7.4.5). Locations of key predicted nucleotide binding residues from Tullet *et al* . (2014) were identified.

**Multiple Sequence Alignment**

*C. elegans* protein sequences for each AAKG isoform were obtained from Wormbase (Version WS282). The PRKAG1 sequence was obtained from UniProt (UniProt Consortium 2021). Sequences were aligned using Clustal Omega (Version 1.2.2). The alignment was viewed in Jalview (Version 2.11.1.7) and coloured by percentage identity, above a threshold of 62%.

**Protein extraction and Western blotting**

Animals were synchronised by bleach preparation and grown on NGM plates seeded with OP50-1. L4 stage animals were washed off plates in M9 buffer, washed twice to reduce bacterial contamination, and pipetted onto either control plates or plates containing 4.5mM phenformin for 48 hours at 20°C. Western blotting was carried out as previously described (Tullet et al. 2014). In brief: Samples were collected using a Diagenode Bioruptor. Proteins were probed using rabbit phospho-AMPKα (Thr172) antibody (Cell Signaling) and mouse β-actin antibody (Santa Cruz) as a loading control. Blots were developed using Bio-Rad Clarity™ Western ECL substrate and GE Healthcare Amersham Superfilm, then imaged using an Optimax 2010 X-ray film processor. Film images were captured using 16MP smartphone camera, then analysed in LI-COR Image Studio Lite (Ver 5.0).

**Lifespan assay**

*C. elegans* were aged on standard NGM plates, seeded with OP50-1 bacteria treated with 10μM 5-fluro-deoxyuridine. All experiments carried out at 25 ^o^ C. Animals were scored every other day and assumed dead when there was no response after lightly touching the body with a platinum pick. Animals that died from causes other than ageing were censored.

**Brood size assay**

10 L4 stage animals were picked individually onto NGM plates seeded with OP50-1 bacteria and maintained at 25°C for the duration of the experiment. Each day, parent animals were moved to new plates, and the plate containing progeny incubated at 25°C for one day before counting.

## Reagents

**Table:** 

**Strain name**	**Genotype**	**Source/Reference**
N2	Hermaphrodites isolated from the CGC Male strain.	CGC
DR1567	*daf-2(m577)*	CGC
PHX409	*aakg-1(syb409)*	Sunybiotech (CRISPR)
PHX410	*aakg-2(syb410)*	Sunybiotech (CRISPR)
PHX411	*aakg-3(syb411)*	Sunybiotech (CRISPR)
JMT61	*aakg-1(syb409); aakg-2(syb410); aakg-3(syb411)*	This Study
JMT48	*aakg-4(tm5539); aakg-5(tm4052)*	This Study
JMT74	*daf-2(m577); aakg-1(syb409); aakg-2(syb410); aakg-3(syb411)*	This Study
JMT43	*daf-2(m577); aakg-4(tm5539); aakg-5(tm4052)*	This Study
